# The very same thing: Extending the object token concept to incorporate causal constraints on individual identity

**DOI:** 10.2478/v10053-008-0119-8

**Published:** 2012-08-21

**Authors:** Chris Fields

**Affiliations:** Santa Fe, New Mexico, USA

**Keywords:** episodic memory, action planning, binding, medial temporal cortex, posterior parietal cortex, autism spectrum disorders, Alzheimer’s disease

## Abstract

The contributions of feature recognition, object categorization, and recollection of episodic memories to the re-identification of a perceived object as the very same thing encountered in a previous perceptual episode are well understood in terms of both cognitive-behavioral phenomenology and neurofunctional implementation. Human beings do not, however, rely solely on features and context to re-identify individuals; in the presence of featural change and similarly-featured distractors, people routinely employ causal constraints to establish object identities. Based on available cognitive and neurofunctional data, the standard object-token based model of individual re-identification is extended to incorporate the construction of unobserved and hence fictive causal histories (FCHs) of observed objects by the pre-motor action planning system. It is suggested that functional deficits in the construction of FCHs are associated with clinical outcomes in both autism spectrum disorders and later-stage stage Alzheimer’s disease.

## INTRODUCTION

Everyday life constantly challenges us not only to categorize the objects we encounter, but also to re-identify some things that we see as being the very same individuals that were encountered in previous perceptual episodes. Re-identifying something - one’s car, for example, or one’s spouse - as the very same individual that was encountered on previous occasions clearly involves both a felt sense of familiarity and a recollection of specific features and context, the two components of the standard dual-process model of recognition (reviewed by Diana, Yonelinas, & Ranganath, 2007; Eichenbaum, Yonelinas, & Ranganath, 2007; Yonelinas, 2002; Yonelinas, Aly, Wang, & Koen, 2010). On this standard model, recognizing an object as the same individual encountered previously involves reactivating an individual-specific representation, termed an *object token*, in association with an episodic memory of the previous encounter. As defined by Zimmer and Ecker (2010), *object tokens* are “what” pathway representations, implemented in perirhinal cortex within the medial temporal lobe (MTL), that bind features specific to and hence diagnostic of a recognized individual to categorical features of that individual. For example, one’s object token for one’s car binds features specific to one’s car - its license-plate number, identifying dents or scratches, personal items carried within it - to the categorical features of its make, model, color, style, etc. as well as categorical features of cars in general. Reactivating an object token produces a feeling of familiarity with the individual object; reactivating an object token in the context of an episodic memory enables recognition of the individual object as the same thing that was previously encountered in the remembered context (Zimmer & Ecker, 2010). Object tokens thus correspond to the individual “items” in the binding of items and contexts (BIC) model of recognition as a coordinated function of multiple MTL areas (Diana et al., 2007; Eichenbaum et al., 2007; Ranganath, 2010; Yonelinas et al., 2010). Object tokens provide an anatomically-specific functional model for the long-term memory (LTM) resident “singular concepts” (Rips, Blok, & Newman, 2006) or “singular files” (Bullot & Rysiew, 2007) that have previously been proposed as explanations of the ability to re-identify individuals (*re-identify* will be used throughout for individuals to avoid the ambiguity between individual and categorical *recognition*).

While the object token concept and the BIC model are well-supported by laboratory studies of feature-driven object re-identification (Diana et al., 2007; Eichenbaum et al., 2007; Ranganath, 2010; Yonelinas et al., 2010; Zimmer & Ecker, 2010), they are challenged by experimental and observational studies of object re-identification in situations involving significant featural change over time, alterations in perceptual context, the presence of similarly-featured distractors, or combinations of such confounding factors. False-memory studies, for example, demonstrate reactivation of object tokens in association with the wrong episodic memories (reviewed by Henkel & Carbuto, 2008, and by Mitchell & Johnson, 2000). Change-blindness studies demonstrate both insensitivity to ordinarily-diagnostic individual-specific features and mis-identification of individuals in the presence of distractors (reviewed by Rensink, 2002; Simons & Ambinder, 2005; Simons & Rensink, 2005). Experiments specifically testing the criteria used to re-identify individuals across perceptual encounters despite featural change and competition from similarly-featured distractors indicate the importance of appropriate causal histories linking the current encounter to previous ones (Frazier & Gelman, 2009; Gutheil, Gelman, Klein, Michos, & Kelaita, 2008; Hood & Bloom, 2008; Rips et al., 2006), the importance of different causal, featural, and categorical criteria to the re-identification of different kinds of individuals (Rhemtulla & Hall, 2009; Rips et al., 2006; Xu, 2007), and the importance of continuity over time of psychological characteristics in the specific case of tracking the identities of individual human beings (Nichols & Bruno, 2010). The human use of causal histories of objects to resolve ambiguities about individual identity introduced by featural change and similarly-featured competitors is well-documented in the anecdotal and philosophical literature (e.g., Bullot, 2009; Bullot & Rysiew, 2007; Nichols & Bruno, 2010; Rips et al., 2006; Scholl, 2007). These diverse results all suggest that a complete account of object-token re-activation and episodic memory retrieval must include an explanation of how causal criteria constrain the re-identification of individual objects across perceptual episodes.

Based on a review of available experimental, observational, and neurocognitive evidence, the present paper proposes that causal criteria constrain object re-identification by a specific mechanism: the construction, by the pre-motor system, of a causal history linking a retrieved episodic memory to the currently-perceived situation. Because the actual histories of objects between perceptual encounters are unobserved, such constructed causal histories are fictive. It is proposed that fictive causal histories (FCHs) play a role in object-token reactivation across perceptual episodes analogous to that played by trajectories in object-file construction within a perceptual episode (reviewed by Fields, 2011b; Flombaum, Scholl, & Santos, 2008; Scholl, 2007; Treisman, 2006): A currently perceived object is considered to be the continuation through time of a previously perceived object only if an appropriate FCH can be constructed. In the case of object file construction, the constraints that define trajectories consistent with object continuity through time are feature-independent (Gao & Scholl, 2010) and are applied within the approximately 50 ms required for visual short-term memory (VSTM) consolidation (Vogel, Woodman, & Luck, 2006). In the case of object-token reactivation, the constraints that define FCHs consistent with the continuation of a previously perceived object between contexts appear to be both category- and individual-specific, and are applied well after VSTM consolidation, in parallel with episodic-memory recall. By proposing that object re-identification depends on the specific mechanism of pre-motor FCH construction, the present model supports the general framework of “embodied cognition” in which the pre-motor manipulation of modality-specific representations implements conceptual inference and problem solving (reviewed by Barsalou, 2008; Kiefer & Pulvermüller, in press).

The next section, *Background*, first reviews four experiments (Brady, Konkle, Alvarez, & Oliva, 2008; Eichenbaum et al., 2007; Guthiel et al., 2008; Simons & Levin, 1998) that illustrate object re-identification under different circumstances. It then briefly reviews neurocognitive evidence for fronto-parietal activations consistent with pre-motor involvement in episodic-memory retrieval (Cabeza, Ciaramelli, Olson, & Moscovitch, 2008; Moscovitch, 2008; Ranganath, 2010; Wagner, Shannon, Kahn, & Buckner, 2005). The third section, *The BIC-FCH model*, describes the extension of the BIC model to incorporate obligate FCH construction. It shows how the extended model accounts for common features of object re-identification that are not explained by the BIC model alone. The fourth section, *Relevance to pathology*, discusses potential clinical presentations of either atypical or disrupted construction of FCHs. It suggests that variant or deficit FCH construction may be detectable in some apraxias, and may underlie common symptoms of both autism spectrum disorders (ASD) and later-stage Alzheimer’s disease.

## BACKGROUND

Consolidation of an object file in VSTM initiates feature-driven object categorization. High-level or super-ordinate categorization (e.g., *animal* vs. *non-animal*) requires less than 200 ms (Kiefer, 2001; Thorpe, Fize, & Marlot, 1996) to approximately 250 ms (Macé, Joubert, Nespoulous, & Fabre-Thorpe, 2009) from stimulus onset, with more specific, entry-level categorization (e.g., *dog* vs. *cat*) requiring at least 50 ms longer (Macé et al., 2009; Martinovic, Gruber, & Müller, 2009). Experiments using fragmented images of animals and everyday objects that require completion to enable entry-level categorization reveal top-down effects from approximately 200 ms, suggesting that entry-level categorization of such images requires at least 50 ms after initial visual processing has been completed (Schendan & Maher, 2009). Categorization times for familiar types of motions are comparable to entry-level categorization times for types of objects: Temporal-lobe cell populations that respond specifically to motions such as pointing or grasping a coffee cup are activated within 200 ms from stimulus onset (Mukamel, Ekstrom, Kaplan, Iacoboni, & Fried, 2010; Tkach, Reimer, & Hatsopoulos, 2007), consistent with response times for the detection of task-relevant motions such as karate attacks observed in athletic events (Mori, Ohtani, & Imanaka, 2002). Adults can recognize point-light walker displays as distinct from scrambled displays within 100 ms (Pavlova, Birbaumer, & Sokolov, 2006), indicating that categorical motion criteria are applied in parallel with static featural criteria, not afterwards, during the categorization process.

The fundamental question that must be addressed by any account of individual re-identification follows directly from the rapidity with which perceived objects are categorized: It is the question of how individual category members that have been seen before are distinguished from individual category members that have not been seen before. Most members of any given entry-level or even subordinate category - most people of a given age, sex, and ethnic group, or most cars of a given make, model, and style - have never been encountered, and their individual features are unknown. A few members of some categories have been encountered before, and their individual features when previously encountered may be accurately represented by one or more LTM-resident object tokens that can be reactivated in association with episodic memories of the previous encounters. If it is assumed that object tokens are reactivated based on featural similarity - producing the “feeling of familiarity” - one possible solution to the object re-identification problem is to use “Leibniz’s law” as a default, identifying any object that is featurally indistinguishable from a reactivated object token as the same individual as the one represented by that object token. Leibniz’s Law embodies two implicit assumptions: (a) that individual, that is, non-categorical features as well as categorical features remain constant over time, and (b) that the probability of encountering two objects with the same individual features is small. Use of Leibniz’s Law as a heuristic will result in identification errors, therefore, in cases involving significant featural change or identically featured competitors. Given that individual features do change, and featural competitors do sometimes appear, human beings can be expected to employ re-identification criteria that go beyond Leibniz’s law. The human use of such criteria has been documented, in various ways, by numerous experiments.

### Four experiments examining re-identification criteria

Experiments in which subjects are required to re-identify objects using only images stripped of meaningful contextual cues illustrate both the power and the weakness of purely-featural re-identification. In the study of Brady et al. (2008), for example, subjects were first presented with 2,500 images, each showing a commonplace object against a white background. They were then presented with a pair of such images, only one of which had been in the training set, and asked to determine which “object” they had seen before in a time-limited, forced-choice design. One-third of the pairs showed images of completely dissimilar objects (“novel” pairs in Figure 1 of Brady et al., 2008), one-third showed images of objects in the same basic-level category (“exemplar” pairs), and one-third showed images of the same object in two different states, for example, a telephone with the receiver on or off the hook (“state” pairs). The frequency with which subjects made the “correct” choice - that is, identified the very same *image* that they had seen before - in the state-pair trials (87%) was statistically indistinguishable from the frequency of correct choices in the exemplar-pair trials (88%). These results suggest that subjects were basing judgments of object identity on whether the images themselves, not the objects depicted, had identical features. If employed in the real world, requiring identical image features to re-identify objects would routinely fail; people employing this criterion would treat objects that had changed state as novel individuals. Hence the results of Brady et al. (2008) indicate that criteria other than identity of image features must be employed to re-identify real-world objects through time.

**Figure 1.**
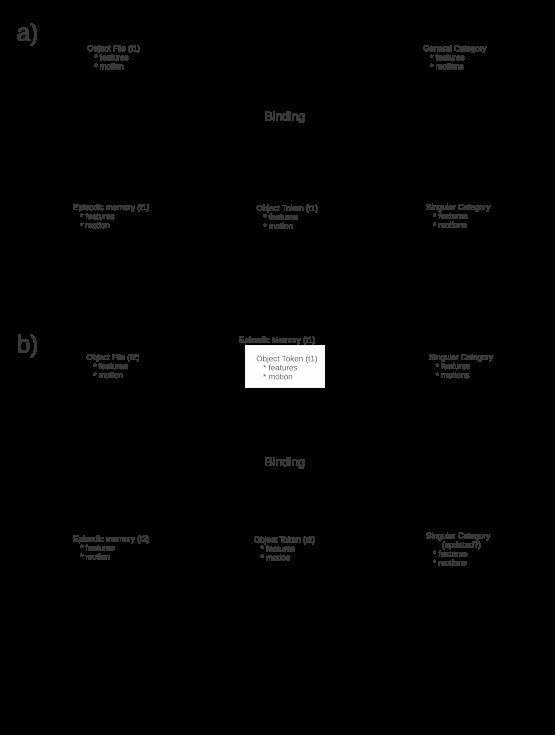
Components of a categorization-based model of individual re-identification. A. Binding of an occurrent object file representing a novel individual results in the encoding of three distinct representations, all of which capture the occurrent features and motion of the novel individual: a timestamped episodic memory representing the event in which the novel individual is participating, a timestamped object token representing the occurrent state of the novel individual, and a new “singular” category. B. Binding of an occurrent object file representing a familiar individual results in the encoding of a timestamped episodic memory representing the event and a timestamped object token representing the occurrent state of the individual. The singular category representing the individual may be updated to incorporate altered features, or may accumulate exemplars depending on the details of the model. The notions *t1* and *t2* represent timestamps.

Experiments probing change blindness provide a complement to studies such as Brady et al. (2008) by examining the effects of small feature changes in an information-rich context. The classic experiments of Simons and Levin (1998), for example, examined the ability of subjects to notice a change of conversation partner - ordinarily a significant event - in the “real life” context of a busy campus sidewalk. More than half of the subjects tested (8/15 in one experiment and 8/12 in another) failed to notice the change, despite obvious differences in the facial features and clothing of the conversation partners. In this rich experimental context, category consistency appeared to be sufficient for re-identification of the conversation partner as “the very same thing” after an occlusion so brief (1 s) that significant changes would not ordinarily be expected to occur. Simons and Levin (1998) remarked that “the fact that we do not expect one person to be replaced by another during an interaction may contribute to our inability to detect such changes” (p. 648). These results suggest that while object tokens may include individual-specific features, these details are in some cases ignored by the object re-identification process in favor of spatial and contextual information shared by two segments of a briefly-interrupted perceptual episode.

The use of manufactured objects presented in three dimensions, instead of as images, provides a means of assessing individual re-identification in the presence of identically-featured competitors. The experiments of Guthiel et al. (2008) employed pairs of identically-featured plush toys representing fictional characters such as Winnie-the-Pooh. Both children and adults were required to determine which of two toys had witnessed and hence “knew about” an action by a child subject. In each trial, the two toys involved were provided with different causal histories by different experimenters carrying the objects in and out of the room where the actions and observations occurred. Over 90% of adults and 80% of children correctly identified the toy that had witnessed the action, even after it had been taken out of the room and an identically-featured competitor introduced. As with the experiments of Simons and Levin (1998), these results show that what an object does dominates what it looks like as a criterion for re-identification as a known individual. They suggest, in particular, that the featural information included in the object token is supplemented by information about the causal history of the represented object.

In ordinary life, human beings are often faced with a combination of featural and contextual changes, as well as separations of hours, days, or even years between perceptual encounters. A commonplace example is provided by the thought experiment with which Eichenbaum et al. (2007) begin their review of evidence supporting dual-process models, in particular the BIC model:

Imagine an occasion when you are walking across campus and see someone who seems vaguely familiar. When she greets you, you are quite sure you know this person, and yet you cannot recall when you met her or why you know her. A casual conversation ensues and you search for clues with innocuous questions. Further embarrassment is avoided when she says something about a meeting last week. Suddenly you recall her name, where the meeting was, and some of the topics discussed there. (p. 123)

In this scenario, the feeling of familiarity is produced immediately, but re-identification occurs after a considerable delay during which additional information is obtained. When re-identification does occur, it occurs suddenly in association with recall of an episodic memory. The BIC model explains the delayed but sudden recall as a consequence of the retrieval, in association with the recalled episodic memory, of a small number of object tokens that are then evaluated on the basis of encoded features to select the best fit to the current object. It does not specify the extent to which current and remembered features of an object must match to produce a re-identification.

Taken together, available data indicate that human beings re-identify a currently perceived object as the same thing as a previously perceived object if the current object is the best “causal continuer” of the previous object (Flombaum et al., 2008; Rips et al., 2006; Scholl, 2007). The current object may have different features than the previous object and it may appear in a different context, but both the featural and contextual changes must be consistent with categorical constraints that specify what kind of object it is and how the features, locations, and contextual roles of objects of that kind can change. This level of sophistication in object re-identification suggests an inferential mechanism more similar to a “mental model” (Gentner, 2002) than to a feature-matcher employing a variant of Leibniz’s law.

### Neurocognitive implementation of object categorization

Objects are typically encountered, and the advantages of accurate re-identification typically arise, in rich contexts involving goal-directed actions. Such contexts are represented by event files, transient bindings of object files representing localized, categorized, static, or moving objects with goals and action plans (reviewed by Hommel, 2004). Categorized object files bind modal image information representing the object as currently perceived with typical feature information represented in lateral and medial areas of the fusiform gyrus (LFG and MFG) for animate and inanimate objects, respectively, and typical motion information represented primarily in superior temporal sulcus (STS) and medial temporal gyrus (MTG) for animate and inanimate objects, respectively (reviewed by Fields, 2011b; Mahon & Caramazza, 2009; Martin, 2007). Active goals and action plans are represented by the fronto-parietal “praxis network” including areas of parietal, cingulate, and both lateral and medial frontal cortex (Culham & Valyear, 2006; Johnson-Frey, Newman-Norland, & Grafton, 2005; Martin, 2007). Unimodal event files are bound in 240 to 280 ms (Zmigrod & Hommel, 2010), the same time-frame required for entry-level categorization. Scenes containing localized, categorized objects are accessible to consciousness after approximately 270 ms (Sergent et al., 2005), suggesting that the event-file level of correlated neuronal activity corresponds to the “global workspace” proposed as the basic substrate of conscious awareness and attentional control (Baars, 1997; Dehaene & Changeaux, 2004; Dehaene & Naccache, 2001).

The primary mechanistic claim of the BIC model is that hippo-campus (HC) binds context and spatial setting information encoded by a “where” pathway involving parahippocampal cortex (PHC) with categorized significant objects encoded by a “what” pathway involving perirhinal cortex (PRC) to encode episodic memories of significant events, and that these same representations are reactivated when the episode is recalled (Diana et al., 2007; Eichenbaum et al., 2007; Ranganath, 2010). Independent data indicating that PRC and PHC are active as components of “where” and “what” perception and imagination (Bird & Burgess, 2008; Graham, Barense, & Lee, 2010; Murray, Bussey, & Saksida, 2007) support this claim. As object tokens are PRC-encoded records of “what” particular objects participated in an encoded episode (Zimmer & Ecker, 2010), reactivation of an episodic memory reactivates the associated object tokens in PRC. The feeling of familiarity with an object requires activity in PRC (Eichenbaum et al., 2007; Zimmer & Ecker, 2010), consistent with involvement of PRC in both the encoding of categorized objects into episodic memories and their retrieval as object tokens associated with episodic memories.

Reactivation of episodic memories is known, however, to involve reactivation of modality-specific representations in temporal cortex (Kiefer, Sim, Herrnberger, Grothe, & Hoenig, 2008; Kosslyn, Thompson, & Ganis, 2006; Moulton & Kosslyn, 2009; Ranganath, Cohen, Dam, & D’Esposito, 2004; Trumpp, Kliese, Hoenig, Haarmaier, & Kiefer, in press; Wheeler, Petersen, & Buckner, 2000) as well as broad activation of parietal and frontal areas (Cabeza et al., 2008; Moscovitch, 2008; Ranganath, 2010; Wagner et al., 2005) in addition to medial temporal lobe; episodic memories contain not only both episode-specific and categorical “what” and “where” information but also information about “how” and “why” objects came to be where they were in a specific recollected context. Experiments that demonstrate reactivation of feature-location, object-motion, and target-action bindings present in recent events (Hommel, 2007; Keizer et al., 2008; Spapé & Hommel, 2010) suggest that entire event files are reactivated by episodic-memory recall. The BIC model as presented does not directly address the incorporation of “how” or “why” information into episodic memories, and hence does not address the question of how target-action bindings are accessed by HC-mediated binding processes. As shown below, this question is resolved by extending the BIC model to incorporate FCHs constructed by the pre-motor system.

## THE BIC-FCH MODEL

### Functional description of the BIC-FCH model

On both the object token model of Zimmer and Ecker (2010) and the BIC model (Diana et al., 2007; Eichenbaum et al., 2007; Ranganath, 2010; Yonelinas et al., 2010), an encounter with a novel salient object A in a context sufficiently significant to be recorded as an episodic memory generates a PRC-encoded representation, an object token, that records the category-irrelevant, individual-specific features of the categorized object file representing A. This object token may be reactivated during a subsequent perceptual encounter in a different context, producing a feeling of familiarity with an object B present in the new context, and possibly a re-identification, accurate or not, of B as the very same thing as the previously-encountered object A. Experimental data as well as common experience indicate that the individual features associated with an object token are applied with different stringencies in different contexts, and that some individual features are more diagnostic of object identity over time than others. Peoples’ faces, for example, are more diagnostic of individual identity than their clothing, and are generally treated as such. Which individual features of the members of a given category are most likely to be individually diagnostic is an item of categorical knowledge. It is useful from a functional perspective, therefore, to refine the BIC model by considering the diagnostic features of an individual to form an individual-specific “singular category” - a category that is presumed by default to have only one member (cf. Rips et al., 2006, who refer to this representation as a “singular concept”). As illustrated in Figure 1 (Panel A), such a singular category is generated, in parallel with the object token, when a novel member of a known category is encountered. Whether the representation of singular categories is anatomically distinct from the representation of object tokens is unknown.

The functional role of the singular category becomes clear when the second encounter with an individual represented by an object token, such as described in the scenario of Eichenbaum et al. (2007) quoted above, is considered. It is critical for successful re-identification that only the diagnostic features composing the singular category are employed as re-identification criteria; otherwise re-identification could be blocked by non-diagnostic features such as style of dress. It is, on the other hand, clear that object tokens, as records of individuals as they appeared in a previous context, contain such non-diagnostic details; otherwise it would be impossible to recognize that someone was dressed differently from before. If the currently perceived individual is a sufficiently good match to the diagnostic features encoded by the singular category to permit re-identification, a new object token capturing the individual’s appearance in the current context is generated. Panel B of Figure 1 illustrates this process.

The frequency with or conditions under which singular categories are updated remains an open question. It is clear, however, that they are updated; otherwise human beings would not be re-identifiable across their lifespans. Singular category updating appears in at least some cases, such as that of human beings, to involve over-writing of no longer diagnostic features with new ones, as opposed to the simple addition of new diagnostic features that may conflict with existing ones. The exactness with which individual features included in a singular category must be matched to enable re-identification, and how the degree to which features must be matched exactly depends on context, is also unknown. Human re-identification abilities clearly impose some minimal-similarity constraints under ordinary circumstances; otherwise it would be impossible to avoid falsely re-identifying someone as a participant in a recallable episode. It would be impossible, for example, to conclude from an examination of one’s memory that a person who claimed to be present during a particular episode was in fact not present. People routinely employ category-specific expectations about featural similarity, assuming that unused artifacts of the same make, model, style, and color will be featurally indistinguishable, for example, but that human beings, animals, other natural objects, and old or used artifacts will be featurally unique (cf. Bullot, 2009; Rips et al., 2006; Xu, 2007). People also treat some features as more “essential” and hence diagnostic than others (Xu, 2007). Extending the object-token framework to include such distinctions does not, however, resolve the question of why stringent feature matches are required for object re-identification in some contexts but not in others. Indeed the usual deployment of category-specific expectations about featural uniqueness exacerbates the re-identification problem; objects such as human beings that are assumed to be featurally unique are nonetheless routinely re-identified despite significant featural changes, raising the question of whether individual features play a primary role in re-identification (Hood & Bloom, 2008; Nichols & Bruno, 2010; Scholl, 2007).

**Figure 2.**
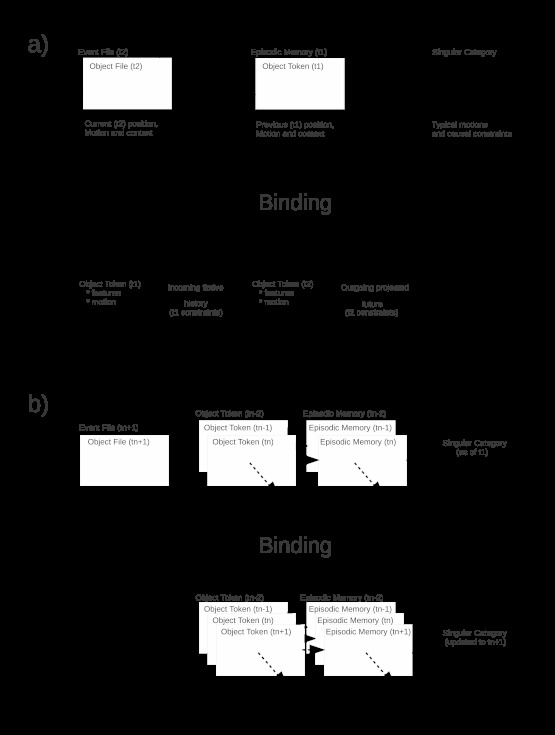
Incorporation of fictive causal histories into a categorization-based model. A. Binding of occurrent object and event files to a previous (timestamped t1) object token, associated episodic memory and associated singular category generates a new object token (timestamped t2) linked to the previous object token by an FCH and extrapolated forward by a projected future. Both interpolation and extrapolation are based on the motion and action constraints available in the singular category as it enters the binding process, that is, the motion and action constraints it encoded as of t1. B. Binding an occurrent object and event file to existing linked lists of object tokens and associated episodic memories appends current (timestamped tn+1) object tokens and episodic memories to the linked lists and updates the feature and motion information in the singular category as required by the current object and event files.

The first hypothesis of this paper is that the model shown in Figure 1 is insufficient to account for human re-identification capabilities, as it provides no mechanism by which the causal history of an object can constrain its re-identification in a novel context. Representing the unobserved causal history of an object between perceptual encounters requires the construction of an FCH. The extended “BIC-FCH” model of object token encoding that incorporates FCH construction is illustrated in Figure 2. The result of encountering a novel object is no different from that shown in Panel A of Figure 1: A singular category comprising its individual features is instantiated when its object token is bound as a “what” component of an episodic memory. Reactivation of individual as well as categorical features on a second encounter generates a feeling of familiarity as described above. Re-identification of the object on a second encounter, however, requires both matching the individual features encoded by the singular category and constructing an FCH linking the previous object token, reactivated in association with an episodic memory, to the current object file. Figure 2 (Panel A) illustrates this more complex re-identification process. The FCH that is constructed must be consistent with categorical constraints on the possible motions or actions of objects in the relevant category (Scholl, 2007; Xu, 2007). A car encountered in the rental-car lot after flying to Europe will not, for example, be identified as the same car one left at home in the U.S., even if it is indistinguishable on cursory inspection from one’s car in the U.S. One is similarly unlikely to identify someone encountered on a university campus as a familiar colleague, regardless of featural resemblance, if one’s last encounter with one’s colleague was 30 min ago, via a phone call to Antarctica. Under normal circumstances, cars do not cross the Atlantic for business trips, and colleagues cannot travel from Antarctica to the U.S. in half an hour; such “normal” facts about how objects can and cannot move are encoded by categorical motion constraints. While encountering an object that exactly or nearly exactly matches the individual features of some remembered object in a context that creates conflicts with categorical motion constraints can produce surprise, under most circumstances resolution of such conflicts does not require conscious deliberation: Causal constraints simply trump featural similarity, just as trajectory continuity trumps featural discontinuity in short-term object-persistence studies (e.g., Flombaum et al., 2008). The encoding of FCHs can, therefore, only require information that is available to the binding process over the time-course of episodic memory encoding, that is, information encoded by the current event file, the previous object token and its associated episodic memory, and the singular and general categories instantiated by the object.

The addition of FCHs to object tokens converts a timestamped sequence of object tokens, as illustrated in Figure 1 (Panel B), to a linked list of object tokens as illustrated in Panel B of Figure 2. Linking object tokens by FCHs links the episodic memories to which they are bound into a historical sequence of episodes: a “life” of the re-identified individual. Such “lives” provide a persistent structure to the singular category upon which annotations of feature changes and post-hoc inferences of context-dependent, individual-specific behavioral regularities can be based, enabling the singular category to serve as an inferentially productive “model” of the individual. The implementation of implicit object models by linked lists of exemplars is typical of event-oriented spatio-temporal database systems, which have substantially greater query-answering capability than earlier, timestamped-exemplar “snapshot” systems (reviewed by Pelekis, Theodoulidis, Kopanakis, & Theodoridis, 2004). In the case of human individuals, models of behavioral tendencies have been shown to be important enablers of re-identification across both radical featural change and causal dis-continuity (Nichols & Bruno, 2010).

### Neurocognitive implementation of fictive causal histories

The BIC model as presented is concerned with HC-mediated binding of context and spatial setting information encoded by PHC with categorized significant objects encoded by PRC (Diana et al., 2007; Eichenbaum et al., 2007; Ranganath, 2010). As noted above, however, the events that are encoded as “context” within PHC are represented as they happen by activations extending across the temporal, parietal, and frontal lobes. A primary function of these extended activation patterns is the planning and execution of context-appropriate goal-directed actions affecting one or more perceived objects. An action plan is effectively a prediction that a represented sequence of transformations will generate a goal state from an observed or imagined base state (reviewed by Bubic, von Cramon, & Schubotz, 2010; Schubotz, 2007). The second hypothesis of this paper is that FCHs are action plans that generate the current context in which an object is observed from the context represented by the most recent episodic memory containing a significant individual feature match to that object. Under this hypothesis, an object is re-identified as a previously encountered individual but only if (a) the features encoded in the current object file significantly match those specified by the singular category associated with an object token linked to a previously-encoded episodic memory, and if (b) an action plan involving the object - an FCH - can be constructed that predicts the current observational context from the context recorded in the retrieved episodic memory. The simplest FCH is one in which nothing happens; this FCH is constructed if the current context is no different from the remembered context. FCH can compensate for uncertainty in feature matching, allowing the stringency at which a feature match is “significant” to remain vague and context-dependent. Inability to construct an FCH, on the other hand, would indicate a violation of some categorical constraint on the causal behavior of the object, such as a car crossing the Atlantic on its own. The BIC-FCH model predicts that objects for which FCHs cannot be constructed due to violations of categorical constraints on actions or motions will not be re-identified as known individuals, regardless of the quality of feature matches to retrievable object tokens or the feeling of familiarity that they engender.

Actions that change object features between contexts involve goals and hence agency. Human beings represent the observed or imagined actions of other agents as mirror-system activations in the action planning system (Cattaneo & Rizzolatti, 2009; Gazzola & Keysers, 2009; Rizzolatti & Craighero, 2004). Observed or imagined motions of inanimate objects that are caused by the actions of agents, such as manipulations of tools, are represented as left-hemisphere biased posterior-parietal cortex (PPC) activations (Culham & Valyear, 2006; Lewis, 2006; Mahon & Caramazza, 2009; Martin, 2007). Visuo-motor networks in superior temporal sulcus (STS) and superior parietal lobule (SPL) are involved in recognizing complex (typically animate) and simple (typically inanimate) motion trajectories as components of events (Fields, 2011b; Nassi & Callaway, 2009). Mirror activations within the right-hemisphere temporal-parietal junction (TPJ) area of inferior parietal lobule (IPL) are particularly involved in associating inferred goals and intentions with manipulations carried out by agents (Cattaneo & Rizzolatti, 2009; Rizzolatti & Craighero, 2004). It is hypothesized that FCHs, as plans representing actions by agents that affect their own states or those of other agents or inanimate objects, are represented by activations of these same systems; in particular, STS for motions of agents, SPL for motions of inanimate objects, and IPL for goal-driven manipulations. The model specifically predicts that mechanical motions of inanimate objects are represented in FCHs by activity within SPL, consistent both with observations of mirror responses to mechanical motions (Engel, Burke, Fiehler, Bien, & Rosler, 2007; Schubotz & van Cramon, 2004), the reconfigurability of mirror-system responses by experience (reviewed by Heyes, 2010), and the human tendency to over-attribute agency to inanimate objects (Atran & Norenzayan, 2004; Heider & Simmel, 1944; Rosset, 2008; Scholl & Tremoulet, 2000).

Fronto-parietal activations have consistently been observed during episodic memory encoding and retrieval, but have been interpreted primarily in terms of task-specific but object non-specific attentional modulation (Cabeza et al., 2008; Ranganath, 2010; Uncapher & Wagner, 2009; Wagner et al, 2005) or the imaginative requirements of reinstating a conscious experience of the remembered event (Moscovitch, 2008; Ranganath, 2010). However, a meta-analysis of activation foci for both episodic memory retrieval and attentional effects in the left PPC suggests that object non-specific attentional modulation cannot explain all episodic memory retrieval-related activation, particularly in IPL (Hutchinson, Uncapher, & Wagner, 2009). The hypothesis that FCHs are constructed by the same systems that represent motions and intentional actions predicts activation of both left and right IPL, as well as STS and SPL. Considering “attention” to be the selective amplification of one activation pattern at the expense of competitors (Chun, Golomb, & Turk-Browne, 2011), re-identification of an object is expected to produce object-specific and action-specific activations in these areas similar if not identical to those observed when a subject attends to particular occurrent objects or actions, as discussed in more detail below. Activations in these areas would be expected during both encoding and retrieval of episodic memories, with the specific activation pattern dependent on the kinds of objects for which FCHs are constructed, and the kinds of motions required by those FCHs.

**Figure 3.**
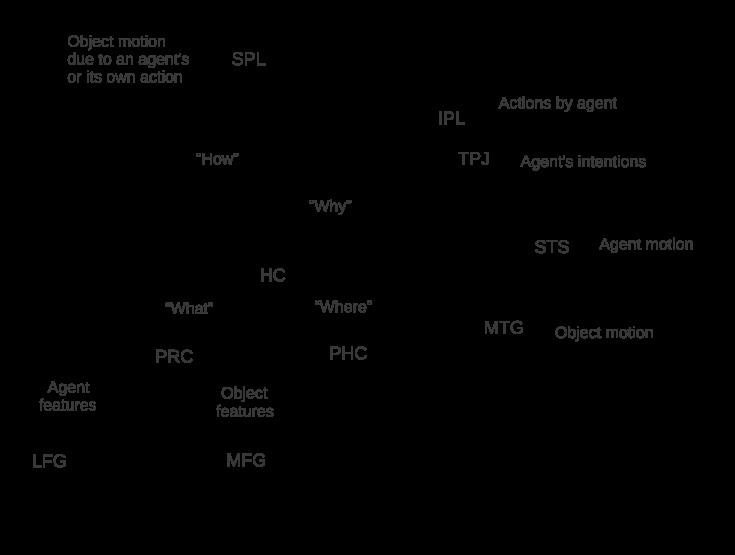
Schematic representation of the temporal-parietal activation loop proposed by the BIC-FCH model. HC = hippocampus. IPL = inferior parietal lobule. LFG = lateral area of the fusiform gyrus. MFG = medial areas of the fusiform gyrus. MTG = medial temporal gyrus. PHC = parahippocampal cortex. PRC = perirhinal cortex. SPL = superior parietal lobule. STS = superior temporal sulcus. TPJ = temporal-parietal junction.

The incorporation of FCHs as implementations of causal continuity constraints extends the BIC model, with its focus on medial temporal lobe (MTL), to the broader BIC-FCH model that couples item-to-context binding in MTL with FCH construction in the superior temporal lobe and PPC. The BIC-FCH model further differentiates familiarity from recollection by adding a temporal-parietal activation loop between feature-driven familiarity and object-driven episodic recollection, as shown in Figure 3. The feeling of familiarity results from activation of PRC-encoded feature representations as predicted by the standard object token concept (Zimmer & Ecker, 2010) and by the BIC model (Diana et al., 2007; Yonelinas et al., 2010). However, many candidate object tokens, and hence many candidate episodic memories, may be activated by the features associated with an occurrent object file. Recognition of a known agent or a known object and hence recollection of a specific previous episode requires the resolution of this ambiguity by the construction of an FCH that links a specific object from a particular previous episode to an object in the current event file by a causal path. Construction of an FCH involves a search for actions capable of mapping a previous “what” and “where” to the occurrent “what” and “where” (Bubic et al., 2010; Schubotz, 2007). Identification of a suitable action answers “why” and “how” an agent or an object could have gotten from the previous episode to the current one, allowing re-identification of the agent or object as a unique, known individual.

As discussed above, the search for an FCH is constrained by both the typical and possible motions and actions that are specified by both the general and individual categories associated with the objects or agents represented in the occurrent object file. For example, an FCH describing the motion of a particular person is constrained both by the general facts that humans typically walk, are able to run, but cannot fly, and by individual-specific facts concerning the particular person’s abilities or preferred gait when walking. These categorical constraints are specialized both to the particular locations and motions represented in the occurrent event file and to the particular locations and motions represented in event files reinstated from retrieved candidate episodic memories. The constructed FCH is an action plan that satisfies both categorical and contextual constraints. The computational complexity of this constraint-satisfaction problem is significantly reduced by the architecture of the action-planning system, which represents actions by force-motion combinations that can be executed by the body (Bubic et al., 2010). In this representation, constraint satisfaction requires coherently scaling the forces and motions used or observed in some previous episode to match the forces available and motions required to produce the goal configuration of agents and objects from the base configuration. Inferences that perform such force-motion scaling are structure mappings (reviewed by Gentner, 2003; Holyoak, 2005; Markman & Gentner, 2001); they are used ubiquitously among vertebrates to perform tool improvisation (Fields, 2011a) and among humans to carry out analogical reasoning in the force-motion domain (Fields, in press). Within the BIC-FCH model, individual re-identification is an effectively analogical process; an individual can be re-identified across perceptual encounters if an FCH can be constructed that is structurally analogous to previously observed or experienced actions.

As indicated in Figure 3, the BIC-FCH model hypothesizes “how” and “why” inputs to HC from SPL and IPL, respectively. These inputs are bound by HC to the “what” and “where” inputs from PRC and PHC respectively to form episodic memories that record not just items and contexts but also actions and the goals driving them. Hence on the BIC-FCH model, episodic memories involving re-identified individuals are expected to have fictive “tails” that correspond to constructed, that is, assumed histories of contexts, actions, and goals. Reactivation of an object token from such an episodic memory would reactivate the context of the remembered episode, but also an FCH of the represented individual that extended back toward previous recallable episodes and forward toward the present situation. Recallable individual histories, however fictive, implement the “models” of individuals illustrated in Figure 2 (Panel B) in the relatively precise functional-anatomical sense defined by Pezzulo and Castelfranchi (2009) and by Bubic et al. (2010). Such models are inferentially productive in that they allow predictive planning based on the anticipation that future goals and actions will be analogous if not straightforwardly similar to past goals and actions.

The BIC-FCH model replaces the intuitive notion that one must sometimes “think about” causal constraints on object identity with the specific, obligate mechanism of pre-motor FCH construction. It thus supports the general theoretical framework of embodied cognition (Barsalou, 2008; Kiefer & Pulvermüller, in press). What it adds to this framework is a process for determining when a reactivated cluster of modal representations refers to the same individual object that it referred to when it was initially encoded.

## RELEVANCE TO PATHOLOGY

The incorporation of FCH construction into object re-identification introduces the possibility that specific functional variants or dysfunctions of the action-planning system may present clinically as specific disruptions in object re-identification abilities. Variant functioning or dysfunction could result in the construction of atypically-precise FCHs that over-constrain object identities, the construction of atypically-imprecise FCHs that under-constrain object identities, or failure to construct FCHs altogether. Functionally-variant FCH construction during infancy would be expected to produce atypical patterns of object re-identification across the lifespan, while deficits due to focal lesions, atrophy, or other insults later in life may be expected to disrupt re-identification only for particular categories of objects or in particular contexts.

If FCHs are constructed by the pre-motor action planning system, one would expect patients suffering ideational or “conceptual” apraxias affecting imagined motions or planning of object-related actions (reviewed by Petreska, Adriani, Blanke, & Billard, 2007) to exhibit difficulties in accurately re-identifying objects in the categorical domains or contexts affected by the apraxia. Apraxics specifically deficient in imagining or planning appropriate uses of tools, for example, would be expected to also exhibit difficulties in re-identifying individual tools, particularly in the presence of context shifts or similarly-featured competitors. Similarly, patients unable to imagine mechanical motions, for example, the motion of a car, would be expected to exhibit difficulties re-identifying objects that execute such motions. On the other hand, patients exhibiting exclusively ideomotor apraxias that disrupt the performance of motor acts but spare action planning and conceptualization would not be expected to exhibit object re-identification difficulties in association with their apraxia; if action planning is spared, FCH construction would be expected to be spared as well. Patients capable of planning and imagining uses of tools but not capable of carrying out the planned or imagined actions, for example, would not be expected to exhibit tool re-identification difficulties.

It has been suggested previously (Fields, 2011b) that insufficient suppression of dorsal-stream trajectory information relative to ventral-stream feature information during early-developmental visual category learning from examples may result in categories that over-emphasize the possible actions or motions and under-emphasize the static features of the categorized objects. A significant over-emphasis on action or motion constraints in early-developing foundational categories could be expected to result in typical outcomes of ASD (APA, 1994), including difficulties in recognizing and developing appropriate emotional attachments to caregivers, delayed and disrupted common noun learning, and low “central coherence” in cognition (Fields, 2011b). The BIC-FCH model extends this suggestion from the domain of perceived motions of category exemplars to that of constructed FCHs. If categorical constraints on actions (for agents or self-propelled objects) or passive motions (for inanimate objects) were atypically narrow, FCH construction would be limited to histories satisfying these narrow constraints. Systematically over-constrained FCH construction could be expected to present clinically as pervasive difficulty in re-identifying objects when they acted, moved, or were moved in ways not previously experienced. In late infancy or early childhood, such difficulties would be expected to disrupt re-identification of family members and other individual human beings, as well as the re-identification of ordinary objects across changes in location or context. ASD patients are known to exhibit specific visual deficits, particularly in the perception of biological motion, the understanding of facial expressions, and the grasping of complex scene gestalt (reviewed by Simmons et al., 2009); however, specific deficits in individual object re-identification across perceptual episodes have yet to be investigated. Experiments that specifically evaluated the object re-identification abilities of ASD patients versus controls matched for IQ and attentional capability, using designs that allowed the experimental manipulation of target object motions and the presentation of target objects in multiple dissimilar contexts, would test the suggestion that ASD involves deficit or variant FCH construction.

Failure to construct FCHs due to disruption or atrophy in the pre-motor action planning system would, on the BIC-FCH model, result in pervasive failure of individual object re-identification, with the types of objects affected dependent on the areas (e.g., IPL vs. SPL) affected by the functional disruption. Specific disruption of FCH construction would be expected to present as a “re-identification agnosia” in which particular individuals could not be re-identified across contexts, even if they could be correctly categorized and both semantic knowledge about the unidentifiable individual and episodic memories containing the individual as a participant were spared. As components of the action-planning system are involved ubiquitously in the management of attention (Corbetta, Patel, & Shulman, 2008), predictive reasoning (Bubic et al., 2010), and self-relevant “default” social cognition (Buckner, Andrews-Hanna, & Schacter, 2008), deficits in individual re-identification could be expected to present in combination with attentional control, planning, and social-cognition deficits. These deficits present in the expected combination in later, demented stages of Alzheimer’s disease, with patients often failing to re-identify family members and other familiar individuals even though they can sometimes recall facts about these individuals and deep episodic memories of contexts in which the unidentifiable individuals participated (reviewed by Jicha & Carr, 2010; Minati, Edginton, Bruzzone, & Giaccone, 2009). Spared deep episodic recall in such cases would be expected not to include “how” and “why” information; whether this is true remains to be investigated. Disruptions of episodic recall have been observed in some patients with non-neurodegenerative posterior parietal lobe (PPL) lesions (reviewed by Cabeza et al., 2008; Olson & Berryhill, 2009); experiments evaluating the preservation of “how” and “why” information in episodic recall in such patients would provide a test the BIC-FCH model.

Category- or context-dependent deficits in FCH construction may underlie some cases of delusional misidentification syndromes (DMS), including Capgras syndrome (reviewed by Feinberg & Roane, 2005). However, the extreme specificity of many DMS cases - for example, the limitation to family members in canonical Capgras syndrome - suggests a primary association with categorization as opposed to causation. The typical involvement of right-hemisphere lesions or atrophy in DMA (Feinberg & Roane, 2005) supports this suggestion, as right-hemisphere areas broadly support semantic, that is, categorical information (reviewed by Bar, 2008), with categorical information about animate objects such as animals and other people particularly biased toward the right hemisphere (Mahon & Caramazza, 2009; Martin, 2007).

## CONCLUSIONS

Ordinary human social life would be impossible without the ability to re-identify individual people, animals, and things across gaps in observation and in the presence of both featural change and similarly-featured distractors (e.g., Dunbar, 2003). It has long been known that human beings employ causal constraints to resolve ambiguity in cases in which re-identification is uncertain (Rips et al., 2006; Scholl, 2007). The implementation of this ability has, however, not been characterized. The present paper proposes a mechanism by which causal constraints can be applied to individual re-identification: the construction of fictive causal histories. It extends the well-supported BIC model of the role of HC in episodic memory encoding and retrieval to incorporate this mechanism. The resulting BIC-FCH model is based on the hypothesis that the pre-motor action planning system constructs FCHs, and hence that a temporal-parietal loop of activation, specifically involving both IPL and SPL, is an obligate component of episodic memory encoding and recall. The BIC-FCH model is supported by both cognitive-behavioral and neurofunctional data, but new experimental designs that specifically control for attentional effects and other potentially confounding factors will be required to test it.

The BIC-FCH model implies that human beings not only assume that objects are persistent through time (Baillargeon, 2008; Scholl, 2007), but that they also assume, via the construction of FCHs, specific unobserved histories for every individual object that they re-identify as being the very same thing as encountered previously. It implies, in other words, that “mental time travel” (Suddendorf & Corballis, 2007) is as post-dictive as it is predictive, that the remembered past - even the past of episodic memories - is as much a cognitive construction as the anticipated future. If the BIC-FCH model proves to be correct, it will show that the cognitive ability to plan manipulations of objects is the foundation on which the assumption of object persistence and hence the possibility of a remembered past are built.
